# The Management of Disclosure in Children’s Accounts of Domestic Violence: Practices of Telling and Not Telling

**DOI:** 10.1007/s10826-017-0832-3

**Published:** 2017-07-28

**Authors:** Jane Elizabeth Mary Callaghan, Lisa Chiara Fellin, Stavroula Mavrou, Joanne Alexander, Judith Sixsmith

**Affiliations:** 1 University of Northampton, Boughton Green Road, Northampton, NN2 7AL UK; 2 University of East London, London, UK; 3 Aristotle University of Thessaloniki, Thessaloniki, Greece

**Keywords:** Domestic violence, Children and young people, Disclosure, Agency

## Abstract

Children and young people who experience domestic violence are often represented as passive witnesses, too vulnerable to tell the stories of their own lives. This article reports on findings from a 2 year European research project (Understanding Agency and Resistance Strategies, UNARS) with children and young people in Greece, Italy, Spain and the UK, who had experienced domestic violence. It explores children and young people’s understandings of their own capacity to reflect on and disclose their experiences Extracts from individual interviews with 107 children and young people (age 8–18) were analysed. Three themes are presented, that illustrate children and young people’s strategies for managing disclosure: (1) “Being silenced or choosing silence?”, explores children and young people’s practices of self-silencing; (2) “Managing disclosures: Finding ways to tell” outlines how children and young people value self-expression, and the strategies they use to disclose safely; and in (3) “Speaking with many voices” considers how children and young people’s accounts of their experiences are constituted relationally, and are often polyvocal. The article concludes that children and young people can be articulate, strategic and reflexive communicators, and that good support for families struggling with domestic violence must enable space for children and young people’s voice to be heard. This is possible only in an integrated framework able to encompass multiple layers and perspectives, rather than privileging the adult point of view. Practitioners who work with families affected by domestic violence need to recognize that children and young people are able to reflect on and speak about their experiences. This requires that attention is paid to the complexity of children and young people’s communication practices, and the relational context of those communications.

## Introduction

The impact of domestic violence on children has largely been studied using quantitative measures of children’s outcomes through questionnaires (generally scored by clinicians or parents/carers) or observations of parent child interactions (Callaghan 2015; Øverlien 2009). This kind of research has documented the harms children experience, including the risk of direct physical harm (Devaney 2008; Jaffe et al. 2012; Sousa et al. 2011), emotional, behavioural and health difficulties (for example, see DeJonghe et al. 2011; Griffing et al. 2006; Holt et al. 2008; Peltonen et al. 2010), relationship problems (Ehrensaft et al. 2003a; Siegel 2013), and educational challenges (Byrne and Taylor 2007; Carrell and Hoekstra 2010).

This literature has played an important role in highlighting the impact of domestic violence on children. However, because of it methodological approach, such research has also been criticised for obscuring children and young people’s own voice (Øverlien 2009). It has been suggested that the failure to talk to children and young people about their lived experiences of domestic violence underestimates their capacity for agency (Callaghan and Alexander 2015; Callaghan et al. 2016b, c; Haselschwerdt et al. 2016; Katz 2015; Mullender et al. 2003; Øverlien 2009) and reproduces a dominant representation of children who experience domestic violence as silent “witnesses”, damaged and damaging because of their “exposure’ to violence” (Callaghan 2015; Eriksson and Näsman 2012; Mullender et al. 2003; Øverlien 2009).

The representation of children and young people as silent and passive is also notable in the policy domain (Callaghan et al. 2016b, c). The Istanbul Convention’s definition of domestic violence shapes most European legislation and policy, and includes “all acts of physical, sexual, psychological or economic violence that occur within the family or domestic unit between former or current spouses or partners, whether or not the perpetrator shares or has shared the same residence with the victim” (Council of Europe 2011). The convention focuses on domestic violence as it occurs in the intimate adult dyad. Elsewhere, this stance has been criticized for positioning children as passive witnesses to adult violence and underestimating the harms children experience (Callaghan et al. 2016b, c). Even where children and young people are not directly physically attacked, the family context of domestic violence is characterized by coercive control and by difficult family interactions that are harmful to children and young people and that violate their right to safety (Callaghan et al. 2016b, c). To understand children’s experiences of domestic violence, it is important to move beyond the physical incident model (Katz 2016), and to recognise the role of coercive control in families—the patterns of abuse characterized not just by violence, but by domination, fear, control, isolation, and degradation (Stark 2007). Framing only the adult in the intimate dyad as “victim”, and reducing domestic violence only to physical violence obscures the interactional context that affects all members of the family, and the harms children and young people experience from both physical domestic violence and coercive control. This adult focused definition also means that children and young people’s own accounts of their experiences are often overlooked in services for domestic violence victims (Callaghan et al. 2016b, c; Katz 2016).

The qualitative turn in social science research on children and young people has resulted in an increased recognition of the importance of hearing children and young people, and respecting their capacity to reflect on their own experiences (Einarsdottir et al. 2009; Skelton 2008). However, research on violence still remains over reliant on adult accounts (McGee 2000; Øverlien 2009). Eriksson and Näsman (2012) have suggested that this emerges from a tension in research and in professional practice between the idea that children and young people have a right to articulate their own experiences, and the emphasis on children and young people’s vulnerability and their right to protection.

Professionals and parents often suggest that children and young people who experience domestic violence find their experiences difficult to talk about. However, as Weiss (2014) has noted, the difficulties people have in communicating violence are often not really about the individual’s inability to articulate their experience; rather the difficulty lies with the listener’s capacity or willingness to listen to the experience being communicated. Whilst it is well documented that adult victims find disclosing domestic violence difficult both because of the emotional impact of the experience, and because of the social stigma associated with such abuse (Liebschutz et al. 2008; Sylaska and Edwards 2013), for children and young people this is exacerbated by professional anxieties about children and young people’s capacity to disclose and their apparent vulnerability when asked about their experiences (Callaghan and Alexander 2015; Eriksson 2012; Eriksson and Näsman 2012). Adult concerns about children and young people’s vulnerabilities and inability to safely reflect on their experiences (often expressed as a worry about “opening a can of worms”, Hester and Westmarland 2005; Lombard 2015) can produce institutionally imposed gatekeeping, obstructions and barriers that result in children and young people’s silencing (Alexander et al. 2016; Skelton 2008) or tokenistic participation (Dexter et al. 2012).

Enabling a space for children and young people to articulate their experiences is particularly important in the context of domestic violence, where not hearing children and young people’s accounts carries additional risks; voices already silenced by violence and coercion in the family can become further silenced by a broader failure to hear what children and young people have to say (Vetere and Cooper 2005). Not listening to children and young people’s accounts of their own experiences of domestic violence can increase the emotional and physical risk to children and young people associated with domestic violence. For example, their accounts of domestic violence might be unheard by parents who are focused on their own coping (Borrego et al. 2008; Katz 2015), or by professionals who privilege the adult or official “story” of the violence (Eriksson 2012). In this way, valuable child protection opportunities can be missed, and opportunities to prevent ongoing family violence can be lost (Buckley et al. 2007). The risk of harm is also evident in contact disputes post-separation, where court orders can override children and young people’s expressions of disquiet about contact with abusive parents (Buckley et al. 2007; Eriksson 2008; Eriksson and Näsman 2012; Featherstone et al. 2013; Hester 2011; Morrison 2015). In addition to such physical risks, professional and parental failure to hear children and young people’s perspective can result in missed opportunities to support them, and to intervene in their emotional and psychosocial worlds (Cooper and Vetere 2008).

Despite the professional, parental and social limitations placed on children and young people that makes articulation of their experiences challenging, a small but growing body of literature has stressed the importance of listening to the experiences of children and young people and of facilitating their voice (Cater and Øverlien 2014; Katz 2015; Mullender et al. 2003; Øverlien 2011). This literature emphasises that children and young people who have lived through violence have a capacity for agency and can reflect on their experiences in ways that challenge their positioning as passive witnesses (Alexander et al. 2016; Callaghan and Alexander 2015; Callaghan et al. 2016a, b, c, d; Cater 2007; Fusco and Fantuzzo 2009; Houghton 2015; Katz 2015; Överlien 2017; Øverlien 2014; Øverlien and Hydén 2009; Swanston et al. 2014). Eriksson and Näsman (2012) suggested that children and young people could reflect on their own experiences of violence, but that talking to them about these experiences required that adults respect children and young people’s right to participation, and balance this against adult concerns about children and young people’s apparent vulnerability. Working with children and young people aged 12–15, Øverlien and Hydén (2009) found they were able to reflect on their own capacity for coping, and describe their own strategies, narrating their lived experience of trauma and of managing trauma. They suggested that children and young people’s personal experiences of violence formed a meaningful narrative, important for their construction of identity. In their study of much younger children (aged 4–7), Evang and Øverlien (2014) found they were able, not only to describe their experiences, but also to take an active role in the form and direction of their interview encounters with the researchers. This enabled children to both manage the interview for themselves, and regulate their own level of emotional engagement when making difficult disclosures. This research highlights that, whilst children and young people may have varying developmental capacities to express themselves verbally (Brooks and Kempe 2012), nonetheless they are able to articulate their experiences and manage interactional spaces across a range of ages.

Evang and Overlien’s work underscores that hearing children and young people’s disclosures of domestic violence involves listening not just to what they say, but how they communicate (Trevarthen 1998), the form of their disclosure, how they perform disclosure differently in different contexts, and how they manage their silences (Callaghan et al. 2015). Failing to understand the performative aspect of children and young people’s disclosures may result in an underestimation of their ability to disclose in a conscious and reflected way. Further, it is important to acknowledge that children and young people’s voices do not occur in isolation, and are not a straightforward reflection of inner experiences—they are interactive, relational, and spatially constituted (Kraftl 2013). Developmental theorists like Vygotsky (1978) and philosophers like Bakhtin (1981) suggested that human beings do not use language merely as an instrument of communication, but that they constitute their sense of self intersubjectively, in dialogue with others. Children and young people’s narratives are consequently is multi-voiced (Bakhtin 1981): children and young people talk to and with others (‘real’ and imagined) when they narrate their experiences. This is an element of the agentic nature of their communications: they tell stories purposefully, in a particular way, for a particular audience and for a particular reason. Attending to the intersubjective and purposeful nature of children and young people’s disclosures is an important but largely overlooked aspect of domestic violence research. Whilst Evang and Øverlien (2014) considered this in the context of the research interview itself, they did not explore how children and young people described their performance and management of their disclosures in a wider relational context. When children talk about their experiences, it is an interactional process (Hermans 2015). The children and young people do not merely recite their stories, but tell them in an interaction that involved the researcher, as well as each child’s own relational history (Evang and Øverlien 2014).

In this paper, we consider children and young people’s reflections on their experiences of disclosing domestic violence. Our aim is to explore how children and young people talk about disclosure, and how they reflect on their experiences and management of disclosure about their family relationships. We highlight their capacity for agency (constrained as it may be by their social and interpersonal contexts), and their careful management of telling, and not telling, of speaking out and staying quiet. This enables us to contribute to a growing body of literature that focuses on children’s capacity for agency in domestic violence, emphasising their capacity to reflect on and manage their disclosures, as conscious, sense-making human beings, rather than as passive witnesses to adult violence.

## Method

### Participants

Child participants were recruited for interview via specialist domestic violence services and refuges or through agencies/practitioners who worked with families affected by domestic violence, such as school family liaison workers, counselling services or children and young people’s hostels. Children and young people were invited to participate if they were aged 8–18, had experienced domestic violence, but were not currently in situations where domestic violence was taking place or were judged by professionals involved with the families to be safe to work with. Although the children of male victims were not excluded, all but two children came from families where their mother was identified by the referring service as the main victim of domestic violence. One hundred and seven children and young people across the four countries participated in semi-structured interviews (see Table 1 for a summary of all children and young people who participated in UNARS interviews, and Table 2 for details of the children and young people whose voices are represented in this article).

**Table 1:** Details of children and young people who participated in the UNARS project

Country	UK	Greece	Italy	Spain
Total participants	21	19	43	24
	Age 8–18 years	Age 10–18 years	Age 8–18 years	Age 11–17 years
Male	13	10	17	8
Female	14	9	26	16

**Table 2:** Details of participants with extracts included in this article

Pseudonym	Gender	Age	Country
Sophia	Girl	15	UK
Nancy	Girl	9	UK
Rachel	Girl	11	UK
Emma	Girl	16	UK
Alison	Girl	15	UK
Ben	Boy	8	UK
Bethany	Girl	10	UK
Matina	Girl	11	Greece
Marios	Boy	14	Greece
Natalia	Girl	15	Greece
Anna	Girl	12	Greece
Lina	Girl	15	Greece
Kostas	Boy	14	Greece
Nacho	Boy	13	Spain
Marta	Girl	17	Spain
Amaya	Girl	17	Spain
Anna	Girl	18	Italy
Elda	Girl	17	Italy
Angelo	Boy	15	Italy
Giacomo	Boy	12	Italy
Age range: 8–18 years			
Mean age: 13.7 years			

The recruitment process was subject to complex access and gatekeeping patterns, and it is consequently challenging to accurately report recruitment rates. In most countries research was carried out in organisations that supported families affected by domestic abuse, and recruitment was always mediated by a third party—domestic violence support workers or similar professionals. The key challenge faced by researchers centred on the gatekeeping practices of professionals. Gatekeeping practices employed by individual professionals and wider organisations make it difficult to know with certainty how many of their clients they actually approached and informed about the research. Gatekeeping also meant that researchers were only given the opportunity to directly inform clients, in person or by telephone, those selected by organisations and considered ‘appropriate research participants’. For these reasons, to report response rates here would be to decontextualize and oversimplify the domestic violence service landscape and culture, producing a potentially false representation of the desires of the family to share (or not share) their experiences.

### Procedure

“Understanding Agency and Resistance Strategies” (UNARS) was a 2 year research project, focused on children and young people in situations of domestic violence. Children, young people, carers and professionals from four European countries (United Kingdom, Greece, Italy and Spain) participated in the UNARS project. The aim of the project was to explore children and young people’s capacity for a sense of agency, resistance and resilience in managing their experience of domestic violence. The data presented in this article focuses on one major emergent theme—that of managing disclosure.

The partner organisations who took part in UNARS shared a common interest in supporting children and young people who experienced domestic violence, and included universities, regional government organisations, social enterprises, and charities. UNARS partner organisations were selected for their expertise in the field of domestic violence and their connections with relevant local agencies. All four countries are signatories to the Istanbul Convention (2011) on Violence against Women and Girls, although Greece and UK are still in the process of ratifying it. The requirements of the Convention mean that all four countries have recently implemented special policies, laws and committed funding to combat domestic and gender based violence at both regional and national level. Despite their socio-cultural, political and economic differences, all four countries have similar reported prevalence rates of domestic violence against women of about 12–15% (Council of Europe 2008). Because of reporting patterns, it is difficult to estimate the number of children and young people who witness domestic violence. However, figures from Greece, Italy and Spain suggest that 63–73% of women who experienced domestic violence had children and young people who had witnessed or heard the violence (Delegación del Gobierno para la Violencia de Género 2015; European Union Agency for Fundamental Rights 2014; Istituto Nazionale di Statistica 2015). Each of these studies focuses primarily on domestic violence with an identified male perpetrator and female victim, and it is likely that this results in an underestimate of the prevalence rates described. A UK prevalence study suggests that 21.9% of 11–17 year old young people, and 24.5% of young adults had experienced domestic violence at some point in their childhood (Radford et al. 2013).

Doing research with children and young people who experienced domestic violence is sensitive and ethically complex. Researchers, carers and referring agencies had concerns that disclosure might in some way endanger children, potentially exposing them to secondary traumatization (see Eriksson and Näsman 2012; Morris et al. 2012) and perhaps put them and their families at risk of repercussions associated with disclosing the violence. Nonetheless, children and young people have reported that they value the opportunity to articulate their experiences, and to give voice to their own capacity for agency (Cater and Øverlien 2014; Houghton 2015). Given this, it was important to find safe ways to facilitate their voicing of their experiences (Alderson and Morrow 2011; Skansvors 2009; Skelton 2008; Valentine et al. 2001).

Researchers ensured potential participants were fully informed about the aim of the research. Children and young people were only recruited if they had left situations of domestic abuse, or if professionals assessed them to be safe to work with (Morris et al. 2012). They informed potential participants that the project focused on domestic violence and explained in detail the procedure and participants’ ethical rights and protections. This information was provided both orally and in writing, before consent was sought. Participants were given time to reflect on this information before the interview was conducted (typically 7 days, but never less than 1 day). Information sheets were written in clear, understandable and jargon-free language and attention was paid to the age of young people involved when presenting this information to ensure that they fully understood what their participation in the study would involve. In particular, it was important that children understood that our focus was on domestic violence, and care was taken to ensure that they understood what this term meant (e.g., by explaining that domestic violence meant families where the adults fought a lot, and where one or both grown-ups would physically hurt each other). Written informed consent was secured if young people were legally able to give it. If not, assent was sought from children, and consent from parents or legal carers. As part of the consent process, we explained to children and young people how their interview data would be used, and explored with them the protections afforded by confidentiality, as well as the circumstances under which it would have to be breached.

Several steps were taken to ensure children and young people’s safety. Anonymity was preserved by using pseudonyms, and removing identifying detail from the interview transcripts and visual material. The research team ensured that information about the study was not taken home by children and young people, and an anonymous contact card was provided to reduce any risk of the perpetrator inadvertently discovering their involvement. Following the interview, the researchers checked with children and young people how they had experienced the interview. If there were concerns about the child, the researchers had access to mental health professionals or domestic violence workers, if consultation or referral were needed.

### Measures

Semi-structured interviews were used to facilitate exploration of children and young people’s experiences of domestic violence. Researchers from all partner countries (ten interviewers in total) attended training to develop the interview materials and to further build skills in conducting interviews with children and young people, ensuring a shared approach to the research. All researchers were educated to at least degree level and were experienced qualitative interviewers. Interviews were conducted in the major language of the region in which the participant lived (English, Castilian, Italian or Greek), and were translated by a bilingual member of the research team. Translations were cross checked against the recording by a second team member.

The interview schedule (see Appendix 1) was designed to be used flexibly, enabling some standardisation across the entire partnership, whilst allowing each country to adapt the schedule to their needs and context. Some examples of the questions used in the interviews can be found in Appendix 1. The interviews lasted between 24 and 83 min and were flexibly structured to meet the developmental level and interactional style of the particular child (Pascal and Bertram 2009). All interviews were audio recorded, and transcribed in full.

### Data Analyses

The interviews were analysed using Denzin’s (2001) *Interpretive Interactionism*, a method that enabled exploration of the intersections of the personal and social in children and young people’s narratives of domestic violence. A descriptive and reflexive summary was produced by each interviewer, using a recording template that formed the front sheet to each interview transcript.

The research team spent 2 days in an analysis workshop, to ensure that there was a common understanding of and systematic approach to the analytic process. In this workshop, researchers worked through the full analytic process together, using transcripts of early interviews completed by each team in each country. Each interview was coded independently, and variations and patterns in coding were discussed and resolved. Virtual team meetings were also held throughout the project (bimonthly), and these meetings included sharing and discussing the emergent analysis. A final workshop was held at the end of the project to agree the major emergent themes. Using extensive field notes and team discussion, researchers maintained a reflexive process to trace the co-construction of the analysis throughout the project (Lincoln and Guba 2005).

The interviews were coded independently by two researchers in each country. Denzin describes this process as “bracketing the phenomenon”, focusing on the participants’ words and expressions as they are in the text of the interview, “cut loose” (Denzin 2001, p. 154) from the world to identify essential features and structures of the experiences children and young people narrated. The researchers compared their initial coding of each interview transcript, exploring variations in coding. Having discussed these discrepancies, researchers agreed a common set of codes for each interview. In the second analytic step, “construction” (Denzin 2001) these bracketed codes are refined by comparing within and then across transcripts, looking for patterns and structures in the interviews, then classifying and ordering them to produce themes. The construction of the themes was completed by the principal investigator (JC) and the lead research associate (JA), in consultation with the full research team. Themes were built to consider both individual variation, and to explore how meanings and experiences were constituted across different children and young people’s narratives within their interpersonal and social context (what Denzin terms “contextualisation”).

## Results

This analysis explores how children and young people reflected on and managed the tensions they experienced around disclosures. Children and young people expressed caution, suspicion and distrust about disclosure, evidenced in the way they talked about disclosing their experiences to friends, family and professionals. Their caution often extended beyond disclosures of violence per se, and into other, apparently more everyday aspects of life. In this sense, speaking out, or indeed, speaking at all, became framed as risky and dangerous. Nonetheless, children and young people were able to find a range of ways to manage their communication with others, and to express what was happening in their families. Our analysis focused on children and young people’s active management of disclosure through strategies of decisive telling and not telling. Children talked about disclosing to a range of other people—to friends, family, and to professionals. This disclosure is managed consciously and agentically by children and young people, and is experienced as a means to protect self and others. Three themes are elaborated. “Being silenced or choosing silence?” explores children and young people’s practices of self-silencing. “Managing disclosure: Finding ways to tell” considers how children and young people value self-expression, and the strategies they use to disclose safely. “Speaking with many voices: Authorised accounts, ventriloquation, and therapeutic talk” details how children and young people’s accounts of their experiences are often polyvocal, and are constituted relationally.

The themes are evidenced with the use of verbatim quotations from interview transcripts in which the child’s name, age, gender and country are provided to give context to the analytical points made. The children and young people whose voices are represented in this analysis all come from situations in which the father (or, in one case, Kostas, the resident paternal grandfather) was the perpetrator of violence within the family.

One of the key elements of the experience of domestic violence for children and young people is that they learn to manage the way they disclose experiences. They learn when to speak out, and when to keep silent, and attenuate their responses to avoid drawing attention to themselves and their family’s problems. Many articulated a sense that they should keep quiet about their experiences, and evaluate the risks of disclosures.

Children and young people were cautious in the way that they spoke about violence itself. Very few of the children and young people interviewed directly labelled violence as violence, preferring instead to use a range of euphemisms or understatements to describe what was happening in their families:

Sophia (15, F, UK): …Something happened to my mum…

Nancy (8, F, UK): the accident scared my mum, and she didn’t like it

Martina (11, Greece): … in all this adventure I told you about…

In each of these examples, prior disclosures by parents and professionals working with the child suggest that the “accident”, the “adventure”, the “something” that “happened” refer to incidents of significant, deliberate and overt family violence. Many of our participants spoke about “arguments” or “fighting”. The interviews were carefully set up to ensure that children and young people knew that they were consenting to speak about their experiences of domestic violence, and domestic violence was explicitly labelled by researchers. This makes the avoidance of labelling all the more remarkable, since interviewer and child both knew that domestic violence had occurred, that the knowledge of that violence was mutual, and that, in that sense, there was nothing to be *hidden*.

So what is being achieved through this kind of euphemistic and avoidant referencing of the very thing they were there to discuss? By attending to the relational and interactional function of children’s disclosures, it becomes clear that children were not just reciting their stories, but were ‘telling’ them to the researcher, narrating their experiences intersubjectively. It appears that their avoidance of the label “violence” accomplishes a range of tasks—it represents the experience in less ‘raw’ terms for the interviewer; it performs a perhaps more child friendly task of enabling them to talk about violence without explicitly labelling it; and it perhaps reiterates the family way of talking about the violence (“having a bit of a temper”, “arguing a lot”, etc., being more common terms used to describe family discord than violence per se). In this analysis, we make sense of children and young people’s accounts of their experiences of disclosing or not disclosing, shifting away from a pathologising account (for example, reading their failure to label their experiences as domestic violence because they are “in denial”) to understand the function of articulating or not articulating, from the children and young people’s point of view.

### Being Silenced or Choosing Silence?

This theme explores how children and young people describe the difficulties of talking about domestic violence. All participants said they felt that they could not talk openly about family matters, and that if they did speak out, they felt they would not be listened to or believed. Their sense of being silenced was in some tension with their sense of actively choosing silence as a way of managing the complexity of familial relationships.

Children and young people reported an attenuation of their speech, a sense of quietening themselves down, to avoid drawing attention to themselves within the family. For instance, Rachel explains how her brother attenuated himself, making his presence less noticeable in response to family violence:

Rachel (11, F, UK): Marcus would like whisper to me and everything because he was scared that he was going to shout too loud or something

Rachel suggests here that her brother has adapted the way that he speaks within his violent familial environment, keeping quiet, avoiding being too noisy. She sees his quietness as a way of keeping himself safe in a risky home.

Elsewhere, we have explored how children and young people’s constant monitoring of their own self-expression functions as an adaptation to the experience of coercive control in the family home—scanning the home environment, tracking adult moods and adapting what they say and how they speak is part of a broader pattern of self-management in a home where drawing too much attention to yourself can be endangering (Callaghan et al. 2016b, c). Some children and young people also reported actual or feared retribution from the perpetrator when violence was disclosed:

Marios (14, Greece): Whatever happened in the family, stayed there. Meaning, if it slipped out, then they ((his family)) would say, don’t know, “why did you tell?” and stuff like that and they would hit you again after. And that’s why it couldn’t be mentioned outside the house.

Here, Marios frames his disclosure, not as deliberate, but as accidental—it “slipped out”. This seems to reflect a family prohibition on speaking about the violence; consequently he needs to defend his disclosure as accidental, as a “slip”. He also frames his subsequent non-disclosure as a non-choice—“it **couldn’t** be mentioned” outside the family. In this way, both disclosure and non-disclosure are positioned as non-agentic: speaking out is a “slip”, being silent is coerced, neither is deliberately chosen. Marios is narrating a kind of agentic double bind; his context makes it difficult for him to actively choose to either speak or not speak. This positioning underscores one of the fundamental tensions children and young people must manage in making decisions about who they speak to and how. The violence and coercion that characterizes the family culture is pervasive, and this has implications for how he is able to make sense of his own capacity for disclosure. The lack of safety means that he must frame any disclosure as ‘accidental’, and that generally he feels that his speech is censured. He has *been silenced*. This is quite different from Rachel’s account of Marcos as choosing to quieten himself.

The risks associated with speaking out may make it more likely that children and young people will maintain a silence that protects family secrets, and that makes disclosure and self-expression more challenging. When asked how she reacted to a very violent incident at home, Anna (18, F, Italy) responded:

I went to school as if nothing happened.

In her description here, it is clear that Anna recognises that she has made a choice (albeit a highly constrained choice) to cover up what has happened at home. She, Marcus and Marios each use a kind of self-protective self-silencing, which on the one hand protects them and their family from the risky consequences of disclosure, but also risks that they do not get the support and intervention they may need.

The young participants also seemed to lack an epistemic trust that they would be heard by adults, and showed a greater awareness that their voices were often discounted in the adult world. Some children and young people reported making explicit decisions not to disclose because of their worries that they might not be taken seriously, or previous experiences of not being believed or of being dismissed. They suggested that being “just children” minimized their social status, and that this meant they would not be taken seriously by adults:

Elda (17, F, Italy): I felt helpless, passive and fragile

Int: What made you feel that way?

Elda: My age

Int: Why?

Elda: It is a constraint. No one listens to you if you’re a little girl

Elda’s experience of helplessness in this extract does not appear to be related to her personal qualities, personal difficulties with self-expression, or some inner state of passivity. Rather, Elda describes herself as constrained by the way she is *viewed* as a child. Her words do not suggest that she sees herself as unable to speak out, but rather suggests that she is constrained by a failure to “listen to little girls”. Her sense that “nobody listens to you if you’re a little girl” positions her as disempowered, fragile and passive. This suggests that children and young people’s positioning as passive and helpless is accomplished relationally; it emerged in Anna’s account as an outcome of feeling unheard. Adults’ failure to hear children and young people’s accounts has consequences for children and young people—it disempowers and isolates them (Buckley et al. 2007). This sense of disempowerment was echoed by Nacho, when he tried to intervene by speaking out to his mother about the violence they were experiencing:

Nacho (13, M, Spain): I told her “Mum, you need help, you need something…” But she told me: “I don’t think so, I don’t need anything else…” I told her “Mum, why don’t you leave this guy? Why don’t you get divorce? Mum, mum, mum…”

Nacho’s intervention here was both courageous and mature—he recognized a need for change, and took action by expressing to his mother his own interpretation of the situation, and his belief that she needed help. His perspective was dismissed by his mother. His words in the extract illustrate the effect of this as his reported voice changes. His voice shifts from mature and adult—“Mum you need help” and “mum why don’t you get a divorce” to a more childlike beseeching—“mum, mum, mum”. The failure to hear his concerns repositioned him as a little child, just like Elda, who “no-one listens to”.

This theme has explored children and young people’s practices of self-silencing—practices that may appear as evidence of the negative psychosocial impact of domestic violence. However, they are also appear to function as complex and adaptive coping strategies that children and young people use to keep themselves and others physically and psychologically safe. Children appear to manage their self-expression in quite conscious ways, managing their self-expression and disclosure. Children and young people showed an awareness of the potential risks involved in disclosing domestic violence, and appeared to make active and conscious decisions to quieten themselves. However, their self-silencing was also achieved intersubjectively, through their positioning as “fragile and helpless”, apparently by adults. Children and young people expressed an apparent lack of trust in adults’ response to their disclosures and this functioned as a significant barrier for children and young people, who reported that it was generally safer to keep quiet about their experiences.

### Managing Disclosure: Finding Ways to Tell

One way children and young people managed disclosure was by finding safe ways to express themselves, and by making clear and conscious decisions about who they could and could not trust with the details of their lives. Despite their sense of disclosure as risky and ineffective, many still seemed to want to talk about their experiences with others. Most described silence as burdensome or difficult, and found safe people, places and ways to disclose.

In the extract below, Natalia suggests that she could not disclose her experiences to anyone, that these experiences were incommunicable:

Natalia (15, F, Greece): I don’t talk about it with anyone else. To whom can I say these things?

The rhetorical form of her statement frames this as an obvious, taken for granted truth—that it is inevitable that she not be able to talk to others about her experiences, that her experiences would not be heard or understood. Similarly, Amaya notes:

Amaya (17, F, Spain): I felt, I felt alone, I have always felt alone, I always felt alone even by being here I felt alone

Amaya’s statement underscores a sense of isolation that this inability to communicate has imposed. This sense of being inevitably “alone” is constituted in familial patterns that produce self-silencing, and that construct their experiences as incommunicable and inexplicable.

Despite this sense of isolation, many children and young people did report having one trusted friend to whom they could disclose. For example, Anna says: friend:

*Anna (12, F, Greece): So from that day on she knows, my friend and I talk to her but she wouldn’t say anything to anyone*

Anna describes cautious management of disclosure. Having weighed up the potential risks of disclosure, she concludes she can safely talk to this individual who she feels would not break her confidence. For many children and young people, this sense of others as trustworthy was built on shared experiences of violence:

Angelo (15, M, Italy): I do not remember, maybe I was talking with my classmate who had the same situation. I was talking with him because sometimes we were at his house or we were at my house and we were talking about these things.

In this extract, Angelo reflects that children with common histories of violence at home were more likely to understand their experiences.

Most participants reported they had one or two trusted confidantes, and these were the only people who they disclosed to.

Lina (15, F, Greece): Anyway and that is, they were amongst the only people I could trust ((.)) but I wasn’t telling them everything, like I was telling Rika, because Rika knew everything about me, whereas the others didn’t know everything about me…. I didn’t discuss family issues, but other stuff with them. They were important, ok, because, sometimes I could find people to talk to, besides Rika… But there’s no need to discuss these with everybody, so ((slight laugh))

Lina describes a highly strategic management of disclosure, consciously selecting friends who she tells about “family issues”, and other friends to whom who she talks to about other matters. She describes a sophisticated relational strategy, recognizing that there are different ways to share with friends, and that she needs to express herself in other ways and other contexts. She assigns specific roles to particular friends to help her manage the expression of her experiences, whilst at the same time managing the risks associated with too many people knowing about her family difficulties.

Emma too expresses caution about who she discloses her experiences to, based in a negative experience of sharing with the wrong people:

Emma (16, F,UK): Yeah, and they ((other children at school)) just found out about it because of, one of, the friend that I told wasn’t the friend that I normally talked to about that kind of thing and I thought I could still trust her anyway. So I don’t have nothing to do with her any more now but, I talked to her about it and then she started telling other people and then that’s how it got round the school kids and I had a lot of problems because of that. They was like, “Ha-ha, your stepdad hates you,” ((mock nasty tone)) and all this stuff.

Emma’s decision to disclose to the “wrong” friend had horrible consequences for her, resulting in continuous bullying at school. She sees talking as risky: it can be misinterpreted, her experiences trivialised, and her disclosures distanced from her own “truth”. She frames her disclosure as an error of judgment, as trusting the wrong person made her potentially vulnerable to further victimisation. This kind of experience (or fear of it) may explain the very conscious decision making that many children and young people reported about who they could and could not trust; the strategic management of disclosure was one of the ways that they kept themselves psychologically and physically safe. Rachel described similar concerns regarding her extended family, who informed her father where she and her mother had fled to, when they sought refuge:

Rachel (11, F, UK): We do see them, we just haven’t like ((umm)) seen them for a while and they were the ones who ((erm)), they sort of like told my dad that we had moved and stuff so we couldn’t really rely on them ((.)) So they knew and they told my dad that we had left…. Yeah, we used to see them quite a lot, we used to tell them quite a lot of things but since then we haven’t really spoken to them.

She and Emma both seemed to conclude that some people are unreliable and unable to keep secrets. They also reflected that keeping secrets was one of the things that helps keep you (and those you love) safe. This is a worrying lesson in some senses, as it entrenches family narratives about keeping violence secret, potentially strengthening coercive and controlling dynamics in the family. By silencing themselves to protect themselves and loved ones, they were also conceding (albeit unwittingly) to the relational conditions that helped to maintain the violence. However, it is important to acknowledge that this secrecy is not straightforwardly imposed on the children and young people—secrecy is not just produced by coercive family dynamics, with the children and young people being passively silenced. Rather these “lessons” are learned in complex relational processes both within and outside the home, and children and young people are active in constructing the family narrative of which they are part.

In addition to their awareness of the physical and psychological risks of disclosure, children and young people were aware that deliberately breaking family secrets was also a powerful thing to do, a gesture of defiance:

Matina (11, F, Greece): However, none of my family knew that me and my sister, we were trusting a common friend that we had.

Int: You didn’t want them to find out.

Matina: ((uh uh))

Matina here described a disclosure of family violence, trusting a friend her family did not even know about. She resisted the family secrecy and silencing with a secret relationship of her own. Disclosure here enables her to safely express resistance to the controlling dynamics of the family.

Throughout the interviews, children and young people showed an awareness that managing their disclosures was a way in which they could assert control and power both in families affected by domestic violence, and in recovery from violence. Despite the risks they perceive, children and young people do talk about their experiences, but this is a complex and fraught process. The examples above illustrate how carefully they weigh up, measure and regulate their decisions about disclosure. This experience can be exhausting and time-consuming. However, managing their disclosures is a means of asserting their capacity for agency, and a form of resistance: they have control over their own story, and how they tell it.

### Speaking with Many Voices: Authorised Accounts, Ventriloquation, and Therapeutic Talk

When children and young people do tell their stories, their accounts are often polyvocal, shifting between adult, professional and child voices as they constitute their narratives. This theme explores the multi-voiced nature of children’s accounts, to consider their implications both for children’s articulation of their experiences of violence, and for our understanding of how children constitute a sense of self in the intersubjective context of domestic violence.

One way that children and young people managed their disclosures was through the production of “authorised accounts”. This refers to accounts that have in some way become official, through repeated telling, or through approval and ratification by adults or others in authority (such as professionals working with the family). The authorised account is typically a neat, and sometimes more sanitised version of children and young people’s experiences. Alison (15, F, UK) referred directly to one version of this:

Alison: If you wanna know my story, fuck off and read my file… Cause it’s all written down ((.)) and that’s one story and people can read the same story. And if it’s written down, you can’t get anything wrong.

“The file” represents the production of a version of family history that is authorised and stable. Variability in the family story is described as necessarily untrustworthy, and Alison is clear that the advantage of producing a single stable written version is that it removed the risk of “getting anything wrong”. In a family characterised by secrets, and frequent court appearances, Alison has learned that if you have to speak about your family, it is best to stick with the authorised version of events, and that “getting things wrong” is dangerous to her and to her family. She has learned to manage her speech very carefully, and seems to have a sense that speaking about her family and her experiences of violence is *risky*. The strength of her concern about getting the version of events “right” is understandable in high conflict families, where there is much contestation of the truth.

Children and young people’s management of disclosure can be achieved in subtle and less subtle ways, drawing on multiple voices to express their experiences. Like “the file”, the family story offered children and young people recourse to an “agreed version” of events, built up within the family. This could sometimes be traced in children and young people’s use of language—perhaps an unusually adult framing of experience, as if they were ventriloquating adult speech.

Int: Who have we got here? ((referring to Ben’s family drawing))

Ben (8, M, UK): The person that ruined my life called Ian ((.)) like I said, he hurt my mum by pushing her down the stairs, and it’s really, and it’s just teared up my family.

Ben’s account here seems to draw on stories he has heard within his family, as suggested by the adult language he used in expressing how the perpetrator “ruined his life”. He seems to draw on a shared language to express shared familial experience that frames the violence and its impact. This illustrates the relational and intersubjective nature of their talk. They do not build their life narratives, or their accounts of violence in isolation: rather these are accomplished in a context, and built up of familial, official and personal accounts of what has happened. Consequently, their stories of domestic violence are polyvocal, reflecting the communally constituted nature of their experiences. It was not simply the case that children and young people were mimicking or repeating adult accounts—but adult stories have been incorporated into their own narratives of their experiences.

Whilst children and young people would very carefully consider and weight up decisions about what to disclose, how and to whom, they do recognize the value of expressing themselves and working through their experiences; they have a sense particularly of the value of therapeutic and supportive disclosures. However, in describing the importance of talking about their experiences, the children and young people again used quite adult constructions of their experiences:

Kostas (14, M, Greece): Every day I was telling more and more, I was taking it out… I was feeling guilty inside of me, I was feeling guilty, how can I say it? And the more I said the more I felt relieved…. I was holding it inside, then I was doing things I shouldn’t do and I was thinking all the time about it, and since then I stopped thinking about it and started talking about it. To somebody.

This idea that “being open” and “talking things out” was a common construction in the recovery accounts of the participants. Talking about difficult things was portrayed by a lot of young participants as a route to healing and recovery, enabling them to put difficult histories behind them. For instance, Marta (17, F, Spain) said:

At that moment I would have liked to have only one person who had believed in me—not even everything I said, you can’t ask so much—but at least someone who supported me. Someone who made that moment of the day a happy one…that…that for a second I didn’t have everything in my head

The burden of self-silencing, and of consequently holding everything inside, and of managing her thoughts and feelings by herself, was expressed poignantly in Marta’s account. Marta’s expressed desire was not just to have someone believe in her, but also to “support” her and provide containment for her, so that she no longer had to carry the weight and complexity of her family history “in her head”. Like Kostas, Marta drew on a therapeutic language to describe and make sense of herself and her emotions.

A quite highly managed expression of experience and feeling is also used by Nancy, in the following extract.

Nancy (8, UK): I talk to this doll, she’s called Nancy as well! And she feels like a real person

Int: She’s like a real person?

Nancy: In a way, she speaks stuff. She’s like a brave doll

Int: She’s brave?

Nancy: She’s like ((puts on an adult voice, American accent)) “You can do it man!”

Int: Does she really speak?

Nancy: In a way yeah. She has a voice recording thing

Int: Oh! ((Laughs)) Okay, so do you talk into the voice recorder?

Nancy: ((Erm)) yeah and it replies. Every time I go up to her and say something, it replies with what I’ve recorded

Int: And what have you recorded?

Nancy: I’ve recorded stuff about being brave, able, able to survive, stuff like that

Int: and how does that help you?

Nancy: It helps me feel like I can make it, I’ll be okay ((.))

Int: What d’you mean “make it”?

Nancy: Like, get to the end of the road

Int: What’s the end of the road?

Nancy: ((errm)) like, happiness

Nancy described how she uses her doll in a range of ways: for example, as a representation of an encouraging, competent and brave possible self—she records her own voice, affirming her own courage which she can then listen to through the doll. In that way, she was able to talk to the doll, seeming to secure support and encouragement through the doll as a proxy self. However, it is important also to look at the kind of voice that she has put into the doll—the voice of an adult, who tells her she can be brave, that she will make it, that she will be OK. In this sense, the doll becomes both a self-project, and a projection of a caring adult, who uses reassurance and encouragement to enable Nancy to cope. Nancy is both coaching herself to better manage her emotions, and using the doll to support her introjection of a positive caring adult, compensating for the relative absence of this care in her own familial relationships. (The use of material objects, exemplified here, is discussed more fully in Alexander et al. (2016).)

This kind of use of very therapeutic talk may be indicative of high service involvement, with children reproducing psychologised ways of seeing and understanding their experiences. Whilst the participants had mostly not had formal therapy, they had often been involved in psycho-educational groups, and traces of their interactional encounters in these groups can be found in their talk. They used terms like stress, release, support, self-esteem—seemingly reproducing the language of professionals, and of various support programmes they had gone through. This kind of language suggests the incorporation of therapeutic dialogue into the sense of self, and the construction of a more managed therapeutic self. This may enable the expression of experience in a way that feels safe and boundaried for children, and that perhaps does not risk too much self-exposure. In reproducing the language of the group they are simultaneously able to express themself, and produce an account of the self that is part of a shared community narrative, authorized by a professional voice.

When considering the sometimes highly crafted nature of children and young people’s accounts described in this theme, it is important not to simply focus on the idea that children and young people are imitating or mimicking adult voices. Rather, children and young people seem to draw on the cultural resources available to them within their families and support services, to make sense of the experiences they have.

## Discussion

When children and young people’s experiences are understood on their own terms, rather than from an adult perspective, it is clear that the dominant image of children and young people as silent witnesses to domestic violence is unsustainable (Callaghan et al. 2016a; Katz 2016; Överlien 2017). What emerges instead is an active, reflexive and strategic decision maker—a child who weighs up all the risks before making a decision about who they will speak to and how. The children and young people interviewed were very articulate, strategic and reflexive communicators. The four themes described above show how their management of what they will and will not say, and to whom they will speak, is a powerful coping strategy, that enables them to establish a sense of being in control of their own (and their families’) life stories. Our study also illuminates the constraints on children and young people’s capacity for articulation, and the ways children and young people work creatively with these constraints. Far from being passive witnesses to violence, the children and young people interviewed had a clear understanding of the interpersonal and social constraints on their ability to talk about their experiences, could reflect on the impact of those constraints, and could find creative ways to work around them.

The analysis explored children and young people’s accounts of disclosure through three interconnected themes. The theme “Being Silenced or Choosing Silence”, illustrated children and young people’s practices of self-silencing—practices that might appear as evidence of the negative psychosocial impact of domestic violence. However, children and young people managed their self-expression and disclosure in ways that seemed quite conscious (see also Evang and Øverlien 2014). They showed an awareness of the potential risks involved in disclosing domestic violence, and appeared to make active and conscious decisions to quieten themselves. However, self-silencing and managing disclosure also appeared to function as complex and adaptive coping strategies that children and young people used to keep themselves and others physically and psychologically safe. This self-silencing was produced interactionally. Participants described how they monitored, weighed up and managed the potential risks of disclosure in domestic violence, making relational decisions about when to speak up, and when to concede to the overt oppressive and coercive behavior of the adult perpetrator. Adult positioning of children and young people as “fragile and helpless”, also functioned to produce silencing intersubjectively. Children and young people expressed an apparent lack of trust in adults’ response to their disclosures and this functioned as a significant barrier for children and young people, who reported that it was generally safer to keep quiet about their experiences.

Neither passive nor silent witnesses, children and young people did value opportunities to discuss their experiences, but they were cautious, strategic and risk focused in their decisions about disclosure (as described in the theme “Managing disclosure: Finding ways to tell”). Much cognitive, emotional and relational work goes into children and young people’s decision making around disclosure and self-expression, as each interaction where disclosure is possible is weighed up by the children and young people. Whilst disclosure is risky (physically, relationally and psychologically), for children, young people and their families, children also recognise that it is a potential resource, that enables them both to gain support and cathartic release.

This management of disclosure underscores the importance of Evang and Øverlien’s (2014) finding that even quite young children were able to manage interview encounters intersubjectively. This analysis highlights that this active management of disclosure in relational encounters is not merely an artefact of the interview interactions, but rather is reflective of a broader relational strategy children and young people use in managing how they tell, and who they tell to. They are skilled in telling their stories, and in avoiding telling their stories.

Previous qualitative studies have highlighted that children and young people have capacity to articulate their experiences (Eriksson and Näsman 2012; Houghton 2015). This analysis has shown that children and young people are highly agentic in their management of this disclosure, but that their ease of interaction with others is limited by their experiences of violence and threat, by their self-silencing, and by their strategic management of disclosure. They have learned to be cautious about what and how they disclose. Further, children and young people’s lack of faith in adult responses to their disclosures acted as an obstacle to disclosure, with many participants reporting that they did not speak to adults because they would not be believed or because they felt that to do so was to risk further harm to self and others.

For fraught and difficult experiences like domestic violence, the available language for children and young people to articulate their experiences can be very limited –culturally available resources to talk about such difficult experiences of family life are very limited. Children and young people’s experiences of domestic violence are often seen as extra-normative, as exceeding what our culture expects of “normal childhood” experiences (Burman 2016). Children and young people’s voices in these contexts may be limited by the symbolic and cultural resources that are available for them to tell their stories, and in this sense their articulation is constrained by the context in which it is expressed (Callaghan et al. 2015; Unterhalter 2012).

Adult talk about violence and recovery is one resource that is available to children and young people. Children and young people’s accounts are therefore multivoiced, with traces of ventriloquation of adult and therapeutic talk in their descriptions. In exploring this polyvocality, the analysis has made visible the intersubjective nature of children and young people’s experiences of domestic violence, and of their construction of a response to that violence. This builds on work that highlights the importance of the relational context in which children and young people experience domestic violence (Callaghan et al. 2016b, c; Cooper and Vetere 2008; Katz 2015; Swanston et al. 2014), extending this to a consideration of how family and professional discourses are used as resources for children and young people to construct their own narratives. Children and young people’s narratives are not built up in isolation, but draw on the cultural and discursive resources that they have access to—just like adult’s narratives.

Recognising that children and young people’s accounts of their experiences are polyvocal, and can at times be couched in very adult terms does not mean that their accounts are unreliable or inauthentic. All human narratives are intersubjective and multivoiced in their nature (Bakhtin 1981). The conscious management of disclosure means rather that children and young people—like all reflexive human beings—are active and reflexive in their production of their own accounts, and consciously and agentically manage their disclosures. It is important to consider the discourses we as professionals make available to children and young people and carers, the language that we use to describe their experiences, because this language takes on a significance for children and young people in making sense of themselves and can act to either enable or block children and young people’s capacity to build a more resilient, resistant and agentic sense of self.

Although children and young people are incredibly resourceful and strategic, the discursive resources available to them in popular culture and professional support to understand and make sense of their experiences of violence too frequently describe them as passive and damaged (Callaghan 2015; Øverlien 2009), positioning them in ways that could disable, disempower and marginalize them, rather than empowering them to cope. For example, professional understandings of domestic violence become an important resource for children and young people who are in contact with services and can help them to frame their experiences. However, if those accounts are framed by normative understandings of childhood and child development, and include entrenched ideas about how violence impacts children and young people, this can have a more problematic impact for the child trying to build a positive image of themselves in recovery (Callaghan and Alexander 2015). In supporting children and young people, it is important to find ways for them to talk about their experiences on their own terms, and to promote less pathologising ways to think about their lives. Children and young people are cautious about talking about their experiences, and consequently will only discuss them in quite specific and limiting conditions. This provides a relatively limited range of cultural resources—often quite adult dominated resources—within which to locate and make sense of their experiences. Recognising how these voices are constituted does not preclude the notion of seeing children and young people’s voices as an expression of their experience. Rather it provides insight into how this experience is constituted.

Consider for instance the way that much domestic violence work focuses on the construct of intergenerational transmission or, as it is popularly framed, “the intergenerational cycle of abuse” (Ehrensaft et al. 2003b; Siegel 2013)—a model that effectively positions children and young people as doomed to repeat cycles of violence and abuse. This construction of children and young people who survive domestic violence offers them two potential subject positions to identify with—future victim or future aggressor. This kind of talk does not equip them with a language register within which to constitute a positive sense of self as survivor, nor does it provide children and young people with an alternative narrative to the family narrative of abuse and victimisation.

Adult and family narratives, and professional discourses about domestic violence can help (and hinder) children and young people articulate an experience that is often difficult to express, both because of a lack of language to talk about violent family relationships, and because of the censoring and self-censoring of their expression. This means that adult and professional language has the potential to facilitate or impede children and young people’s talk about their experiences of domestic violence, and through its shaping influence on children and young people’s narratives may have the potential to impact children and young people’s envisioning of their present and future sense of self. We therefore need to think carefully as professionals about the way that we listen to and talk about (and with) children and young people, ensuring that we use language in ways that support children and young people’s capacity for agency, and that respect how they have coped historically with the violence they have experienced. This requires that professionals working with children and young people and families must interrupt and refigure talk that positions children and young people as helpless witnesses, or that discounts or dismisses children and young people’s accounts.

Adult accounts of surviving childhood domestic violence have identified having someone to confide in outside the home as an important resilience factor (Anderson and Danis 2006; Gonzales et al. 2012). Whilst access to social support is an important aspect of children and young people’s resilience in domestic violence (Anderson and Danis 2006; Gonzales et al. 2012), the act of disclosure also functions as a resistance to an imposed regime of silence—a gesture of defiance that enables children and young people to hold on to a thread of self-determination and a sense of self that is defined beyond the immediate experiences of violence and coercion. In a context where maintaining silence is an element of coercive control, breaking silence is a potent act of resistance (Callaghan and Clark 2007). Children and young people’s accounts suggest that they recognize the potential strategic disclosure has as a form of resistance to the coercive control of the perpetrator parent. It also enables the child to hold an active protective and care-giving role towards the rest of the family, resisting the perpetrator’s definition of the family (Callaghan et al. 2016a, b).

This potentially opens up a window in which the child is able to construct a sense of self beyond the familial patterns of violence and abuse. Disclosing in a safe space, finding routes to safe self-expression can enable children and young people to maintain a toehold of self-determination and a sense of agency in the face of the abusive behavior of the perpetrator parent, thus challenging the apparent totality of the abuser’s control (Callaghan et al. 2016a, b). As Hebdige (1979) suggests, small gestures of defiance signal a refusal, they have a subversive value that extends beyond the immediate act of defiance itself. In the context of domestic violence, such gestures of defiance signal a refusal of the coercive practices and oppressive silences of the family—they are an explicit resistance to the familial order which is characterized by regimes of silence. For children and young people, speaking out, however quietly, signals a holding on to a sense of “me” that enables a distance to be built between them and familial patterns. It enables them to feel they can protect themselves and their families, by making conscious choices about who to tell, how and when they tell.

In common with much research on children and domestic violence, this study was limited by its focus on children and young people recruited through support services for domestic violence. It would be useful to understand how children and young people’s narratives of disclosure are constituted when they are not in families that have been involved with services, and whether the multivoiced shaping of their disclosures is different in a community sample. Further, whilst it was not our intention to only recruit families where the main identified victim was female, only a small number of participants came from families where the main identified victim was male, or where professionals judged both partners to be violent. This limits the relevance of these findings to similar families. A further concern in interpreting the findings of this research is that presumably those children and young people who chose to participate in the study were more inclined to talk about their experiences, potentially skewing findings to children and young people more comfortable with disclosure.

This research emphasises the need for professionals working with children and young people affected by domestic violence in which they can disclose safely, and work on the production of alternate family narratives. One way to enable this is to ensure that all those who work with children and young people affected by domestic violence establish clear lines of communication, in which children and young people know the boundaries, and understand the limitations and potential consequences of disclosures. It is crucial that children and young people are supported to feel secure in their disclosures, so that they can rebuild their sense of trust in adults who both believe them, and are trustworthy. By making boundaries overt and explicit, professionals and other adults can enable relational space in which the pressure for children and young people are relieved of the need to constantly scan and monitor the safety of their disclosures. At the same time, these professionals need to respect children and young people’s strong need to manage their disclosures, to keep themselves and their loved ones safe and therefore must respect children and young people’s (constrained) capacity for agency.

It is important too that professionals listen to children and young people’s accounts, and that children and young people’s narratives are accorded the same respect and sense of credibility as that accorded to adults. It is also important that professionals and other adults supporting children and young people who experience domestic violence overcome their own collusions with the silencing of children and young people. This can be achieved by recognizing that children and young people are conscious, sense-making beings who are aware of the violence they have experienced, and have developed complex strategies for coping with those experiences (Vetere and Cooper 2005). This requires that those supporting children and young people suspend adult denial and minimization of both the impact of domestic violence on children and young people, and of their agentic capacity to cope with and manage that violence and its consequences. Hearing children and young people’s voices means attending not only to what children and young people say, but also to how they say it; this involves learning to listen not only with our ears, but to attend closely to the complex forms of children and young people’s disclosures and to understand and contextualize how it is both constrained and enabled. This requires strong support for families struggling with domestic violence within an integrated framework that can encompass multiple layers and perspectives rather than privileging the adult one.

To provide a respectful and child focused environment for survivors of domestic violence, professionals and other adults need to understand the relational context of children and young people’s lives in which their multivoiced and multi-textured narratives are constituted. This creates an environment in which the co-construction of the family narrative, and children’s conscious and protective management of disclosure is understood in context, and not dismissed as “coaching” or “parroting” of adult perspectives. This requires a safe, flexible, responsive, but clearly structured environment that can encompass multiple layers of disclosure and make sense of its implications. Telling their stories in such a safe and respectful context will enable both professionals and children and young people to make sound decisions that support a sense of rational and realistic maintenance of safety in the family, beyond the coercive relationships that have previously characterised their family life. This will enable the emergence of alternative stories of self and other that could lay the foundations for children and young people to live their life free of abuse.

## Appendix 1

Sample from Interview Schedule

1. Could you tell me a little bit about yourself? For example, where do you come from, do you have any brothers and sisters, where do you live now, and with who?
2. How would you describe your family? If you had to tell the story of you and your family, what would it be?
3. This project is about children growing up with domestic violence—with lots of fighting and maybe hitting in their home. Do you think of yourself as growing up in that kind of situation? What is that like for you?
4. When there were bad times at home, when people were fighting or getting angry with each other, what was that like for you?
5. How do/did you cope with those kinds of situations?
6. Is there anything you did that made you feel better, when bad things were happening at home? What did you do / say? How did it help?
7. Is there someone you can talk to about the things that happen or have happened at home?
8. What do you think needed to change to make things better at home? What could other people have done to change things?
9. How do you think you could have changed things?
